# Effect of Different Coring Thicknesses on Tooth Movement During Processing of Complete Dentures: An In Vitro Study

**DOI:** 10.7759/cureus.106754

**Published:** 2026-04-09

**Authors:** Akshaya Kadunganari, Nandakumar Karamannattil, Aysha Mohamed Ali KP, Neethu Niduvote Poyil, Priyanka K, Shahnaz Shahnawaz

**Affiliations:** 1 Department of Prosthodontics, Crown and Bridge, Muslim Educational Society (MES) Dental College, Perinthalmanna, IND; 2 Department of Prosthodontics, Crown and Bridge, Kannur Dental College, Kannur, IND; 3 Department of Prosthodontics, Crown and Bridge, Royal Dental College, Palakkad, IND

**Keywords:** complete denture, coring thickness, intermolar distance, tooth movement, tooth position

## Abstract

Objective: This in vitro study was designed to assess the impact of varying coring thicknesses on the extent and orientation of tooth movement during complete denture fabrication.

Materials and methods: Standardized maxillary and mandibular complete denture wax-ups were fabricated and divided into four groups based on coring thickness (1 mm, 1.5 mm, 2 mm, and 2.5 mm). Coring was achieved using vacuum-formed thermoplastic sheets of predetermined thickness adapted over the occlusal surfaces. A total of 112 samples (n = 28 per group) were processed using conventional heat-cured polymethyl methacrylate (PMMA) denture base resin. Pre- and post-processing intermolar distances were measured using a digital caliper, and tooth movement was analyzed using one-way ANOVA with Bonferroni post hoc tests.

Results: Tooth movement was observed in all groups; however, a significant difference was noted between the groups. Group A exhibited the lowest degree of tooth displacement, followed by Groups B, C, and D. Decreasing the coring thickness showed a statistically significant reduction in tooth movement, particularly in the anteroposterior (p<0.05) movement.

Conclusion: Within the scope of the present study, it was inferred that a 1 mm thickness of coring was found to be the ideal coring thickness. This suggests that incorporating optimal coring thickness may enhance the accuracy and stability of complete dentures.

## Introduction

Dental polymers possess adequate physical, mechanical, and aesthetic properties as a denture base material [1]. A diverse array of polymers is frequently employed for several uses in prosthodontics. Polymethyl methacrylate (PMMA) is extensively utilized in dentistry for prostheses for the fabrication of denture bases, obturators, orthodontic retainers, and temporary crowns [2]. Denture bases made of PMMA experience dimensional changes during processing and use. It also shows adverse effects, such as linear shrinkage, which can lead to unfavorable tooth movement. These micro-movements can cause significant changes in balanced occlusal forces [3]. A complete denture's quality is impacted by a number of processing factors that could result in base distortion and a subsequent change in tooth position. These variables include the varieties of acrylic and investing medium, the technique for introducing resin, and the temperature at which polymerization is activated [4]. Numerous flasking and polymerization procedures and materials have been researched in an effort to combat these unwanted processing effects. The strength of the investing materials is also responsible for such unwanted tooth movements [5,6].

The processing of resin dentures is highly technique-sensitive and presents significant challenges for both dentists and technicians due to the alterations that take place during denture fabrication. The movement of teeth during processing is possible in the medio-lateral, anterior-posterior, and vertical directions, resulting in occlusal discrepancies that were not present during the try-in stage [7]. The movement of teeth during denture fabrication has been a subject of debate, related to the nature of the materials employed and the processing techniques utilized [8]. The internal stresses produced in acrylic resin dentures during polymerization are alleviated during cooling, resulting in alterations in dimensions after deflasking, decasting, and throughout the finishing process of the denture [7]. The teeth are moved due to the setting expansion of the second pour of plaster investing stone in the flask. Research indicates that this expansion results in tooth movement of 0.02 to 0.05 mm [6,7]. Numerous studies have been conducted to reduce tooth movement during denture fabrication by employing various coring materials for investment; nonetheless, the findings are inconsistent [6-8].

It is suggested that utilizing artificial stone as an investing medium could substantially diminish tooth movement. Variations in the material thickness of the denture base are additionally linked to variations in tooth mobility [6]. Flask closing technique and time after pressing were said to be key elements affecting tooth displacement. Since its inception, PMMA has emerged as the substance used most frequently for denture bases. Excellent aesthetic properties, acceptable strength, reduced water absorption, decreased solubility, non-toxicity, ease of repair, and construction using uncomplicated molding and processing techniques are just a few of the benefits of PMMA [9]. Polymerization shrinkage has an impact on the final occlusion of the dentures. Maintaining the established occlusal framework from the try-in session to delivery has consistently posed a significant challenge in complete denture fabrication [10].

Coring is a procedure involved in investing where a thin layer of type III gypsum is applied over the occlusal and polished surfaces of the waxed denture to reproduce the fine details [8]. To the best of our knowledge, no study has been done relating to the effect of coring thickness on tooth movement during the fabrication of dentures. Henceforth, the present in vitro study aimed to determine the tooth movement in different coring thicknesses (1 mm, 1.5 mm, 2 mm, 2.5 mm) during the processing of a complete denture. Thereby, the adequate thickness of coring material to minimize occlusal discrepancy could be determined.

## Materials and methods

This comparative in vitro study was conducted between January 2021 and August 2022 after approval from the Institutional Ethics Committee (IEC/MES/56/2020). The sample size was calculated using the mean and standard deviation of intermolar distance of a similar previous study [11]. 

The sample size was calculated using the mean and standard deviation of intermolar distance of a similar previous study [11] using the formula: N = ((Z(1-α/2) + Z(1 + β))² × (σ1² + σ2²/r))/(μ1−μ2)², where alpha (α) = 0.05, beta (β) = 0.2, mean of second intermolar distance before processing (μ1) = 48.023, standard deviation of second intermolar distance before processing (σ1) = 0.455, mean of second intermolar distance after processing (μ2) = 47.727, standard deviation of second intermolar distance after processing (σ2) = 0.646, sample taken in each group = 28, total sample size = 112. 

All linear measurements of tooth movement were carried out under identical experimental conditions by a single calibrated examiner using a standardized measuring device in order to guarantee measurement reliability. The mean value was noted for statistical analysis after each measurement was performed three times at different intervals. Twenty percent of the samples were chosen at random and re-measured after a week to evaluate intra-examiner reliability. The intraclass correlation coefficient (ICC) was used to assess the consistency between the two measurements. Excellent repeatability and dependability of the measurement process were indicated by a high ICC value of 0.93. To reduce measurement error and improve the precision of the recorded tooth movement, standardized reference points on the casts and denture teeth were used throughout the study.

A custom-made metal mould was fabricated from an aluminum round metal block with right and left first molars, with definite reference points placed on the mesiobuccal cusp tips of both molars. The metal mould was customized with a 50 mm intermolar distance. This 50 mm intermolar distance was taken from the mean values of the intermolar distance of 20 maxillary complete dentures. The expected tooth movement was compared with the proposed dentures. The dies (n = 112 specimens) were prepared with the customized standard metal mould. Each waxed prosthesis was later invested in Plaster of Paris. The separating medium was applied over the plaster area, and four types of flexible splints were fabricated with thicknesses of 1 mm, 1.5 mm, 2 mm, and 2.5 mm.

The coring thickness was standardized during the fabrication of the flexible splints using a vacuum-forming technique. Thermoplastic sheets (Ethylene vinyl acetate, Erkodent, Germany). Diameter 120 mm of predetermined thicknesses (1 mm, 1.5 mm, 2 mm, and 2.5 mm) were used to obtain the required coring thickness for each group. The sheets were adapted over the waxed prosthesis using a vacuum-forming unit, ensuring uniform thickness across the occlusal surface. The thickness of each fabricated splint was verified using a digital caliper (Generic, Mumbai, India) at multiple points to ensure accuracy and consistency before placement in the mould. Based on the verified thickness, these samples were thus classified into the following four groups: Group A: 1 mm of flexible splint, Group B: 1.5mm of flexible splint, Group C: 2mm of flexible splint, and Group D: 2.5mm of flexible splint.

Each flexible splint was placed over the occlusal surface of the waxed prosthesis. The second and third pours were done with dental plaster, which was then transferred to the dental clamp. After setting of the plaster (beta calcium sulfate hemihydrate, Kalabhai Karson Pvt. Ltd, Mumbai, India), the dental flask was opened, and the splint was removed. This space was filled with dental stone (alpha calcium sulfate hemihydrate (Ultrarock) by Kalabhai Karson Pvt. Ltd, Mumbai, India). Dewaxing was done and separating medium (Dental Products of India Ltd (DPI), Mumbai, India) was applied over the mould space. The heat-cure acrylic resin (poly methyl methacrylate (DPI Heat Cure) by Dental Products of India Ltd (DPI), Mumbai, India), monomer polymer, was added in a ratio of 1:3 by volume. It was then mixed and inserted into the mould in the dough stage. Trial closure was performed after a bench cure of 30 min, followed by processing of the denture prosthesis.

**Figure 1:**
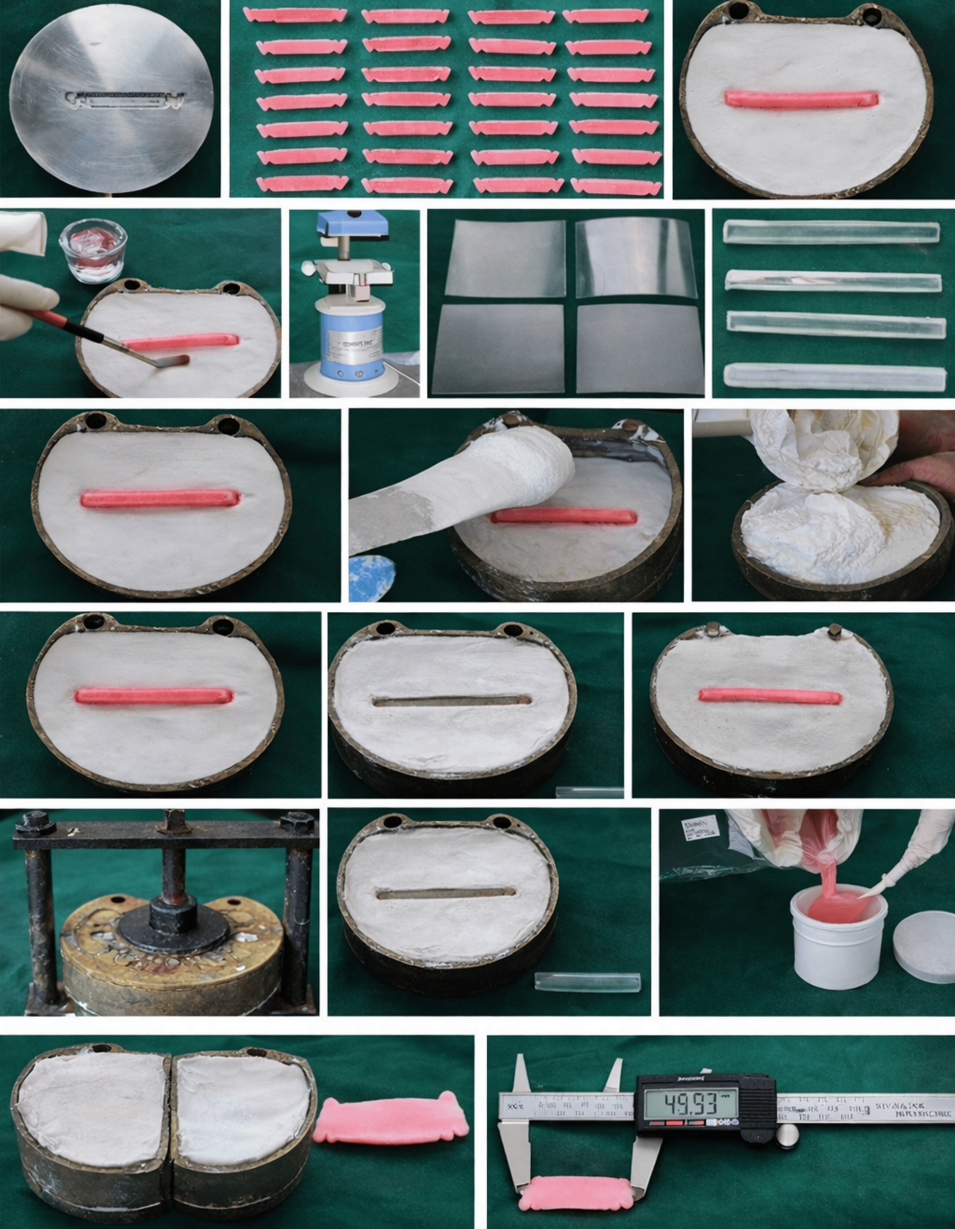
Schematic representation of procedures, including filling of the dental stone, dewaxing, manipulation of heat-cure resin, deflasking, and measurement of the prosthesis using a digital caliper.

Each deflasked denture prosthesis was compared with the customized standard metal mould. Measurements were taken at the same reference points. The readings were tabulated. The readings of the proposed dentures were compared with the customized standard metal mould. The intermolar distance was measured with a digital caliper (Generic) (Figure 1).

Statistical analysis

The statistical software IBM SPSS Statistics 26.0 (IBM Corporation, Armonk, NY, USA) was utilized for the data analyses. The results were presented as mean±SD. A one-way ANOVA was used to determine significant differences between groups. The multiple comparisons were done using the Bonferroni post hoc test. The significance level was fixed at 5%.

## Results

A total of 112 samples were selected. Each group consisted of 28 samples. The samples were divided into four groups as shown in Table 1.

**Table 1:** Distribution of samples in each group. Samples based on flexible splint thickness (Group A: 1 mm; Group B: 1.5 mm; Group C: 2 mm; Group D: 2.5 mm).

Groups	Flexible splint dimension (mm)	n	%
Group A	1	28	25
Group B	1.5	28	25
Group C	2	28	25
Group D	2.5	28	25
Total		112	100

Group A exhibited the highest mean of 48.5±0.8 mm, and the lowest mean value was noticed in group D (46.1±0.6 mm). The groups B (1.5 mm of flexible splint) and C (2 mm of flexible splint) showed an identical intermolar distance after processing (47.6±0.4 mm). The coring thickness was standardized using a soft, flexible splint material, and the data revealed that as coring thickness increases, the maxillary intermolar distance decreases (Table 2).

**Table 2:** Maxillary intermolar distance after processing (mm). Flexible splint thickness (Group A: 1 mm; Group B: 1.5 mm; Group C: 2 mm; Group D: 2.5 mm).

Groups	n		Mean±SD (mm)
	A	28	48.5±0.8
	B	28	47.6±0.4
	C	28	47.6±0.4
	D	28	46.1±0.6

**Figure 2:**
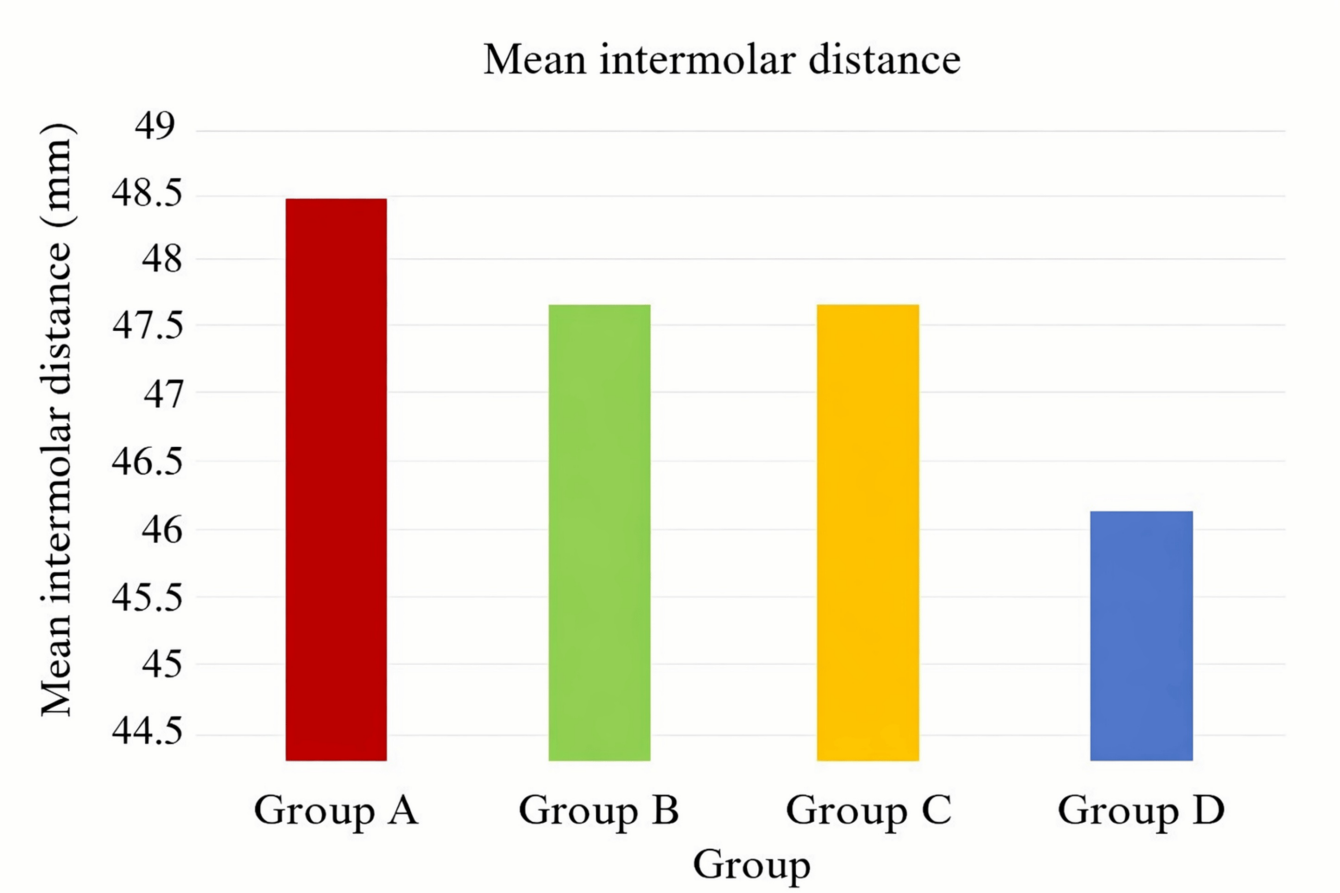
Mean intermolar distance in all groups.

The mean difference in intermolar distance between the four groups was analyzed using one-way ANOVA (Table 3, Figure 2) and was found to be statistically highly significant (p<0.01).

**Table 3:** Mean comparison of maxillary intermolar distance (mm) for four specimen groups using ANOVA. df: degrees of freedom.

Source	Sum of squares	df	Mean square	F-statistics	p-value
Between treatments	79.01	3	26.34	72.21	0.0001
Within treatments	39.39	108	0.36		
Total	118.40	111			

The data were then subjected to multiple comparisons using the Bonferroni post hoc test (Table 4). While the pair-wise comparison of groups B and C revealed a statistically insignificant difference (p>0.05), all other multiple pair-wise comparisons of the specimen groups revealed statistically highly significant differences between the groups (p<0.001).

**Table 4:** Multiple comparisons (post hoc) test of study specimens. **Highly significant. Samples based on flexible splint thickness (Group A: 1 mm; Group B: 1.5 mm; Group C: 2 mm; Group D: 2.5 mm).

Pair-wise comparisons		Mean difference (mm)	95% Confidence interval of the difference		Bonferroni correction statistics	p-value	
			Lower	Upper			
Group A	Group B	0.9	0.56	1.24	5.64	<0.001**	
	Group C	0.9	0.56	1.24	5.64	<0.001**	
	Group D	2.4	2.02	2.78	14.54	<0.001**	
Group B	Group A	-0.9	-1.24	-0.56	5.64	<0.001**	
	Group C	0.0	-0.21	0.21	0.34		0.22
	Group D	1.5	1.23	1.77	8.89	<0.001**	
Group C	Group A	-0.9	-1.24	-0.56	5.64	<0.001**	
	Group B	0.0	-0.21	0.21	0.34		0.22
	Group D	1.5	1.23	1.77	8.89	<0.001**	
Group D	Group A	-2.4	-2.78	-2.02	14.54	<0.001**	
	Group B	-1.5	-1.77	-1.23	8.89	<0.001**	
	Group C	-1.5	-1.77	-1.23	8.89	<0.001**	

## Discussion

The processing of complete dentures entails dimensional modifications that can lead to the displacement of artificial teeth. The current study identified that variations in coring thickness affect tooth movement during denture processing. Several factors work together to cause this effect, including the investment material expanding, the denture base resin shrinking as it polymerizes, and the way stresses are spread throughout the mould during processing.

PMMA, a type of acrylic resin, is still the most common material for denture bases because it is easy to work with, looks good, and has good mechanical properties. However, changes in size that happen during polymerization are unavoidable and are one of the main problems with this material [12]. When PMMA is being processed, it expands when heated, then contracts when cooled, and finally shrinks in volume when polymerized. This can cause stress inside the denture base and the mould around it. These stresses are then released during deflasking, which causes the denture base to become misshapen and the teeth that are embedded in it to move. It has been reported that the linear polymerization shrinkage of heat-polymerized acrylic resin can range from about 0.3% to 0.5%. This can have a big effect on the positional stability of denture teeth during processing [13,14].

The thickness of the coring is very important for controlling how much investment material is around the denture teeth in the flask. During the curing process, investment materials like dental plaster or stone expand as they set [6]. This expansion could push on the teeth that are embedded and cause small changes in their positions while they are being processed. Earlier studies have documented tooth displacement ranging from approximately 0.02 to 0.05 mm due to the setting expansion of the investing material [7]. When the coring thickness is increased, the larger amount of investment material may show more cumulative expansion, which can change or cancel out the effects of polymerization shrinkage. On the other hand, if the coring thickness is not thick enough, this compensatory effect may be lessened, which would allow the resin's polymerization contraction to have a bigger effect on tooth movement [15].

Another important part of the process is how the stresses are spread out in the mould during the polymerization cycle. When heat-curing, the acrylic resin changes from a plastic dough stage to a hard polymer network. This change also causes the volume to shrink, which puts tensile and compressive stresses on the denture base and at the interface between the resin and the investment material. Finite element analyses have shown that higher levels of polymerization shrinkage greatly raise the internal stresses in denture base structures [16]. The thickness of the coring layer affects how these stresses are passed on to the investment material around it. A thicker coring layer helps keep the teeth in their original position by evenly spreading out stresses and making the mould less likely to deform. On the other hand, a thinner coring layer may not be stiff enough to handle these stresses, which could cause the investment to deform in certain areas and the denture teeth to move more [17].

Thermal conditions throughout the curing cycle may also influence the impact of coring thickness on tooth mobility. The polymerization of heat-cured acrylic resin entails temperature fluctuations that can affect both the contraction of the resin and the expansion of the investment. A thicker investment layer can help keep the heat from moving too quickly and help the resin polymerize more evenly. This regulated thermal setting may mitigate sudden stress accumulation within the denture base. On the other hand, a thinner coring layer may allow heat to move quickly and unevenly through the mould, making it more likely that the mould will warp and the teeth will move [14,18].

Significant variations in balanced occlusion are caused by tiny motions. Such unintentional tooth motions are also caused by the strength of the investment materials. The waxed denture's polished occlusal surface is covered with a thin layer of type III gypsum throughout the investing process to replicate the intricate details [19]. Thus, the present study aimed to determine the relationship between various coring thicknesses and the position of teeth after processing, to identify the optimal coring material thickness to reduce occlusal disparity. Studies on the effects of various investing materials on tooth mobility have already been conducted [6,20,21]. To our best understanding, no investigations have examined the effect of coring thickness on tooth movement during denture production.

Pero et al. [22] determined that the thickness of the acrylic resin specimen and microwave polymerization cycles affected porosity. For any thickness, there were no differences in porosity in the polymerized resin bases during the water bath cycle. Mosharraf et al. [23] stated that gradual cooling after processing a heat-activated acrylic denture base helps lower distortion of the prosthesis, even though several processing procedures have been devised to minimize polymerization shrinkage. Numerous flasking polymerization processes and materials have been researched to counteract these adverse processing effects. To enable trial packing without disturbing the acrylic resin of the veneers, a mixture of dental stone is applied over the top of the teeth in the invested trial denture, a process known as "coring" [19].

The stability of denture teeth during processing depends on finding a balance between changes in size that work against each other. The polymerization shrinkage of the acrylic resin tends to pull the teeth toward the resin mass on the one hand. The setting and thermal expansion of the investment material may, however, push the teeth out [24]. The strength of these opposing forces is affected by the type of investing material, the flasking technique, the processing cycle, and the thickness of the denture base. There is a lot of evidence that factors related to how you invest and process can have a big effect on how teeth move in full dentures [6,17].

Based on these mechanisms, coring thickness is an important processing variable that affects how well complete dentures fit. A good coring thickness gives more investment bulk, better stress distribution, and better resistance to the forces that resin polymerization shrinkage creates. These factors all work together to make artificial teeth more stable while they are being processed into dentures. On the other hand, not enough coring thickness can make the mould less structurally sound, which can make denture teeth more likely to move during curing and deflasking.

Gypsum compounds are crucial auxiliary materials utilized in numerous clinical and laboratory processes, even if they are not specifically employed as dental restorations. When compared to other types of investment materials, dental stone remains the superior choice for casting acrylic denture bases in traditional molds and experimental processes [7]. Due to changes that take place during the manufacturing of dentures, processing resin dentures is technique-sensitive and, in fact, a tough experience for dentists and technicians. The least amount of tooth movement during processing was seen when dental stone was employed as the coring material over the teeth and polished denture surface, together with dental plaster. In the processed dentures, a straightforward method of coring with dental stone would lessen tooth motions and improve occlusion. The alterations that take place during the processing of dentures have always been attempted to be minimized [25]. The type of material and the processing method have been suggested as the causes of the movement of teeth that happens during denture construction. The current study also suggests that incorporating optimal coring thickness may enhance the accuracy and stability of complete dentures. Additional clinical studies are warranted to strengthen these findings.

Despite the constraints of an in vitro design, the results of this study underscore the significance of regulating coring thickness during the flasking and investing phases of complete denture fabrication. Optimizing this parameter may help reduce tooth movement, improve occlusal accuracy, and cut down on the need for major occlusal adjustments after processing the denture.

This study has some limitations. It was conducted under in-vitro laboratory conditions, which may not fully replicate the clinical oral environment. The sample size was limited, and only the maxillary intermolar distance was evaluated as the measurement parameter. In addition, only one denture base material and a conventional processing technique were used. Therefore, the findings may not be directly generalized to all clinical situations. Further clinical studies with larger sample sizes and different materials or processing techniques are recommended to validate these results.

## Conclusions

Within the limitations of the present in vitro study, 1 mm coring thickness demonstrated the most favorable outcome among the tested groups and may be considered the most suitable thickness under the experimental conditions employed. It was also observed that coring thicknesses ranging from 1 to 2.5 mm were associated with a reduction in occlusal discrepancy, although the magnitude of discrepancy tended to increase with increasing coring thickness. Therefore, while 1 mm coring thickness appeared to perform best in this in vitro setting, these findings should be interpreted with caution. Further clinical studies are necessary to validate their applicability in routine practice before definitive clinical recommendations can be made.
